# Supplementary figures and images for: Physiological and transcriptomic analyses reveal the cadmium tolerance mechanism of Miscanthus lutarioriparia
Source: PLoS One. 2024 May 15;19(5):e0302940. doi: 10.1371/journal.pone.0302940 (PMC11095687; doi:10.1371/journal.pone.0302940)

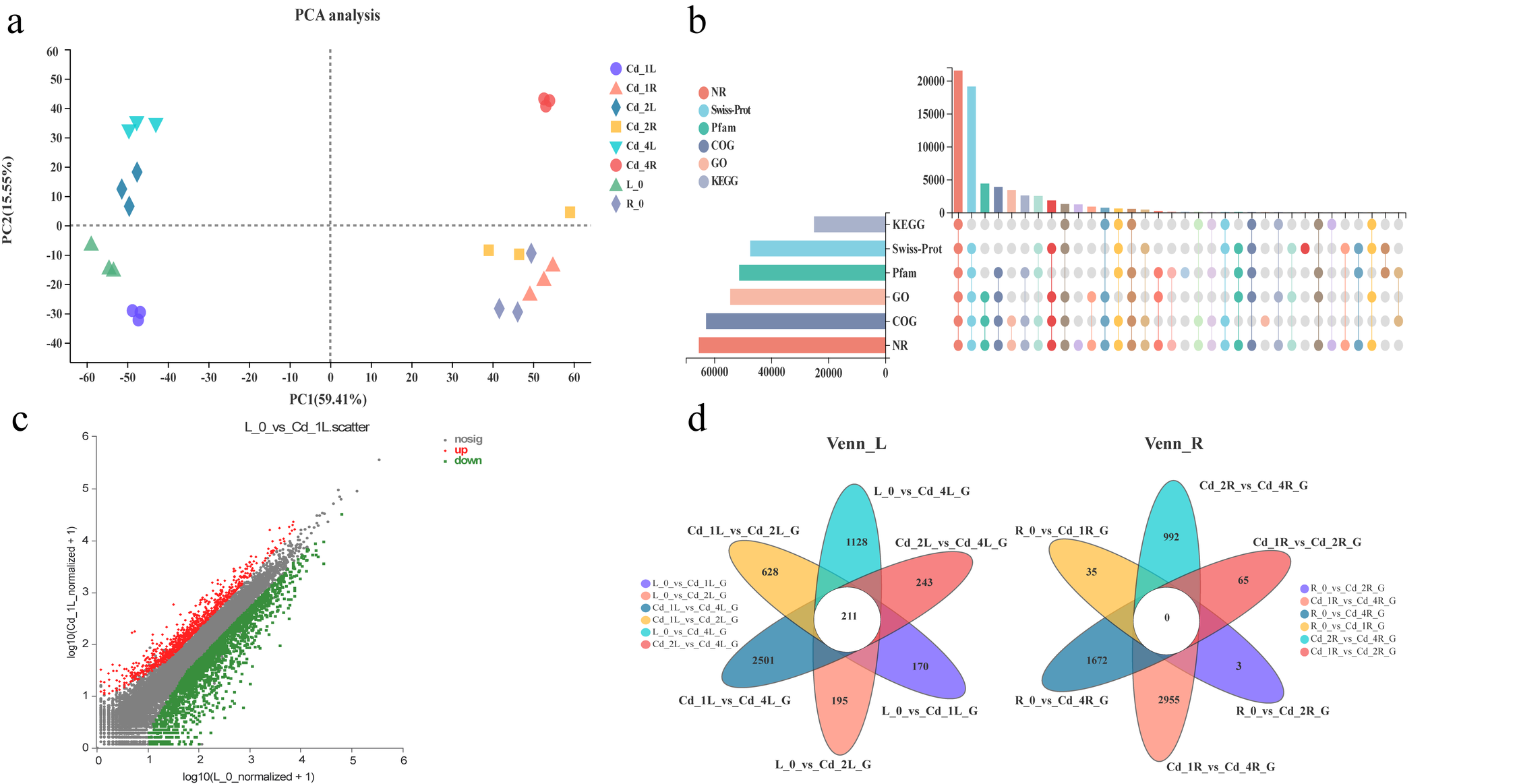

Supplement: S1 Fig — (a) Principal components (PCA) analysis the transcriptome similarity and repeatability between samples. (b) Gene annotation. (c) The analysis of differentially expressed genes (DEGs). The red indicates up-regulated expression of DEGs, while green indicates down-regulated expres-sion of DEGs. (d) Venn diagram of DEGs in different root and leaves samples. L0, 1L, 2L, 4 L, leaves of M. lutarioriparia treated with Cd for indicated periods. R0, 1 R, 2 R, 4R, roors of M. lutarioriparia treated with Cd for indicated periods. (TIF) [file pone.0302940.s001.tif]

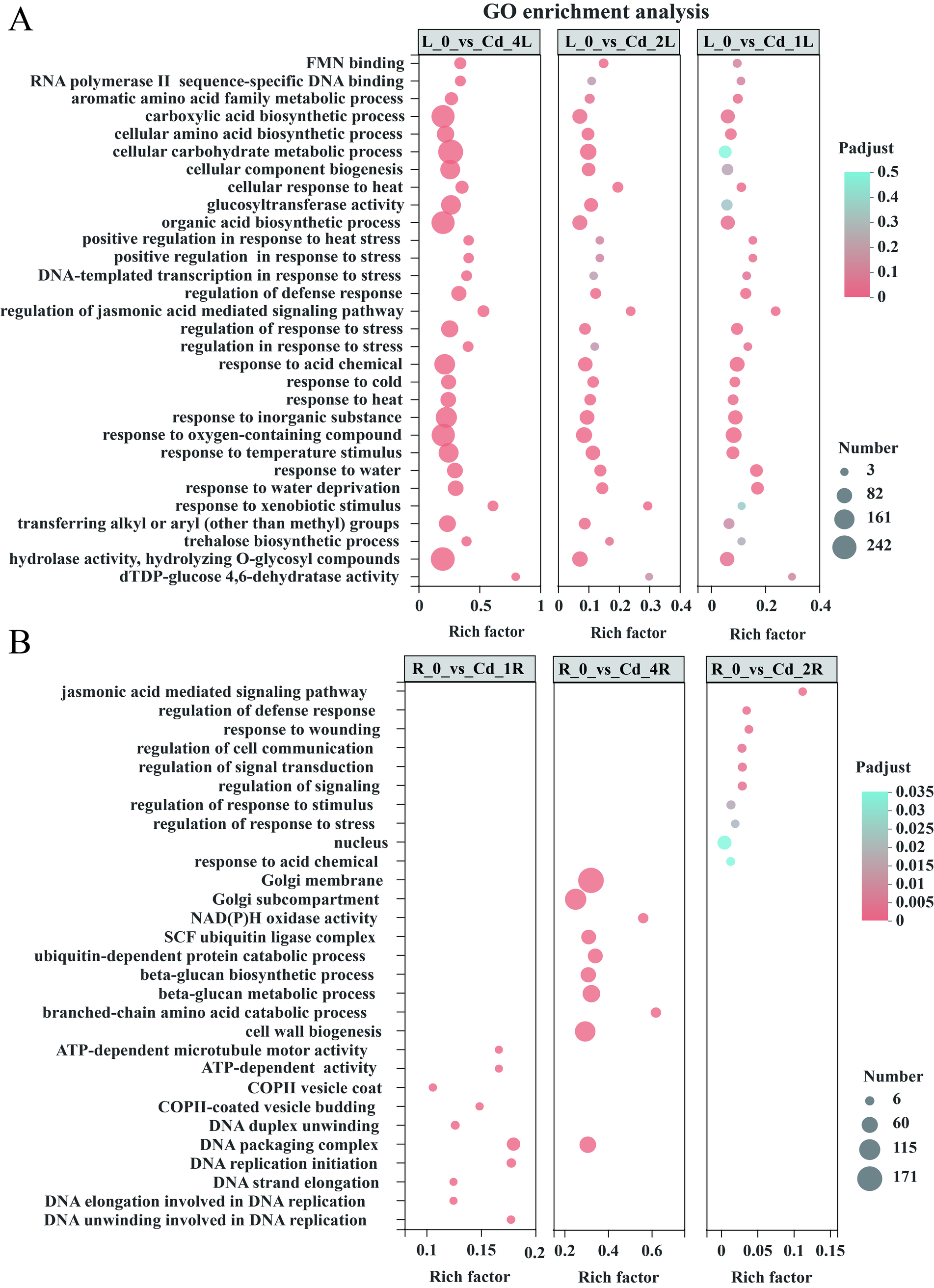

Supplement: S2 Fig — (a) GO enrichment analysis of DEGs in leaves, (b) GO enrichment analysis of DEGs in roots. The x-coordinate represents the enrichment rate, and the y-coordinate represents the pathway.L and R are the leaves and roots, respectively. (TIF) [file pone.0302940.s002.tif]

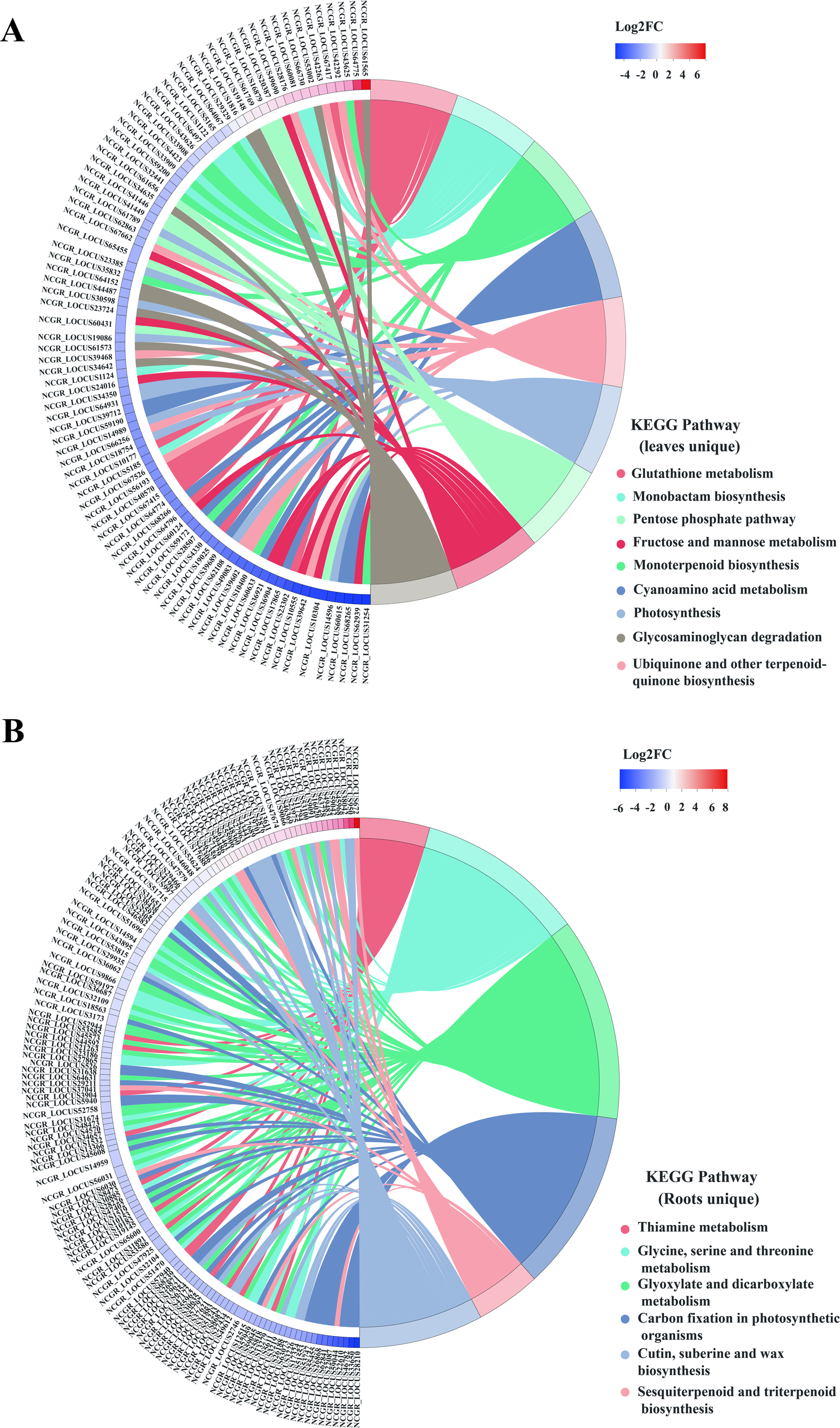

Supplement: S3 Fig — (a)KEGG enrichment analysis of DEGs in unique leaves, (B) KEGG enrichment analysis of DEGs unique in roots. (TIF) [file pone.0302940.s003.tif]

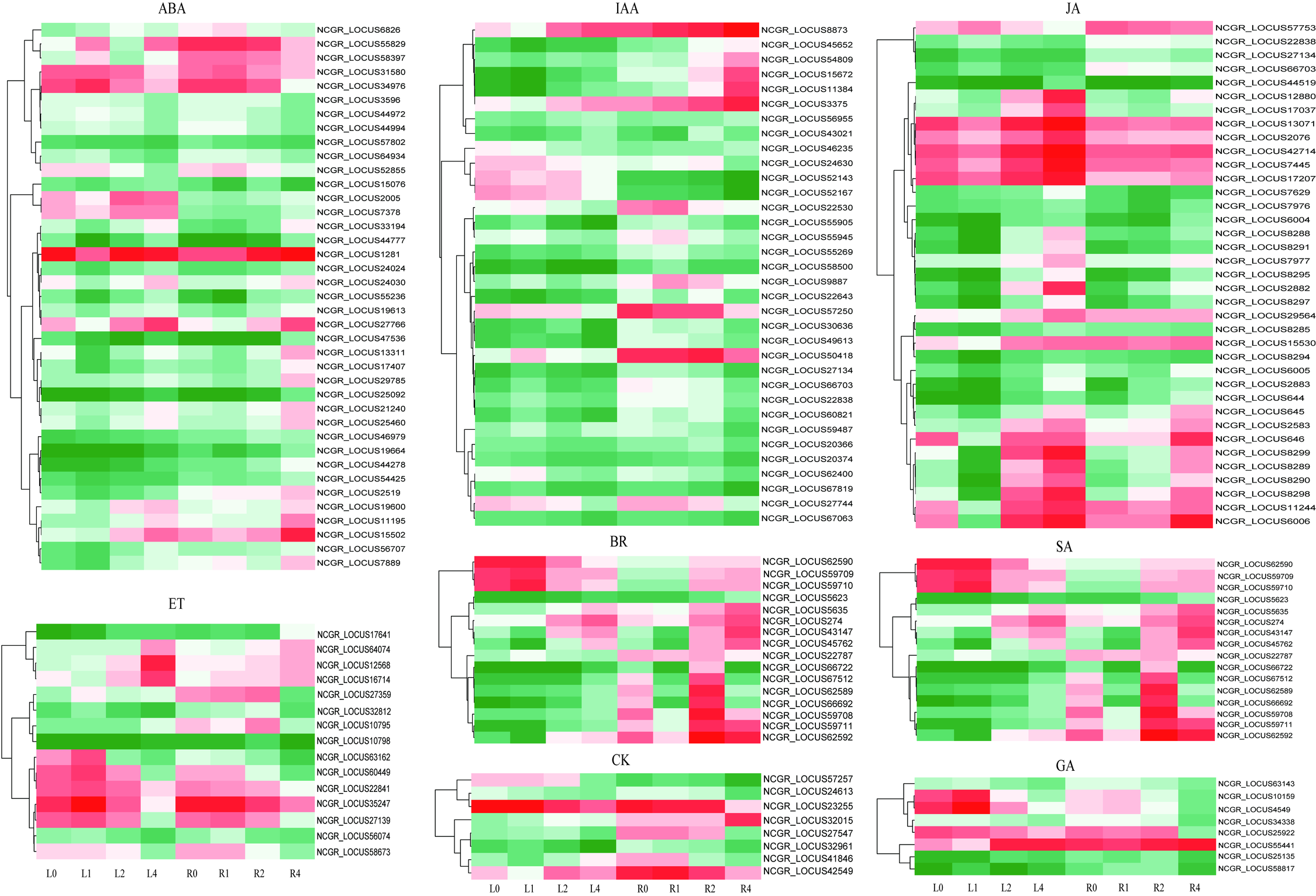

Supplement: S4 Fig — abscisic acid (ABA), ethylene (ET), auxins (IAA), brassino-steroids (Br), Cytokinin (CK), Salicylic acid (Sa), jasmonates (Ja), and gibberellins (GA),. The red indicates up-regulated expression of DEGs, while green indicates down-regulated expression of DEGs. 0d, 1d, 2d, 4d, days of plants treated with Cd for indicated periods. (TIF) [file pone.0302940.s004.tif]

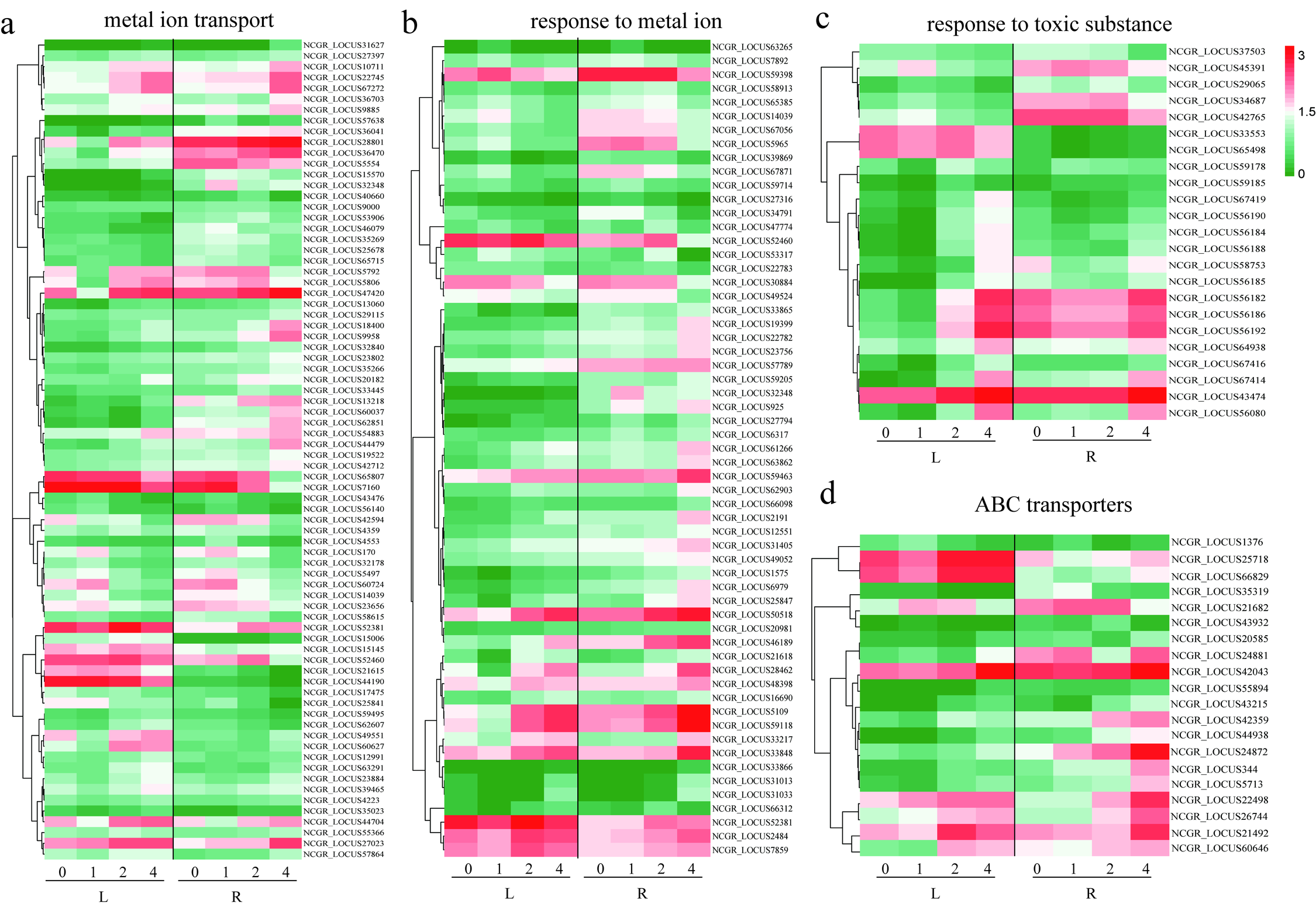

Supplement: S5 Fig — Expression level analysis of metal ion- and transport-associated DEGs under the Cd stress, including metal ion transport (a), response to metal ions (b), response to toxic substances (c), and ABC transporters (d). The red indicates up-regulated expression of DEGs, while green indicates down-regulated expression of DEGs. 0d, 1d, 2d, 4d, days of plants treated with Cd for indicated periods. (TIF) [file pone.0302940.s005.tif]

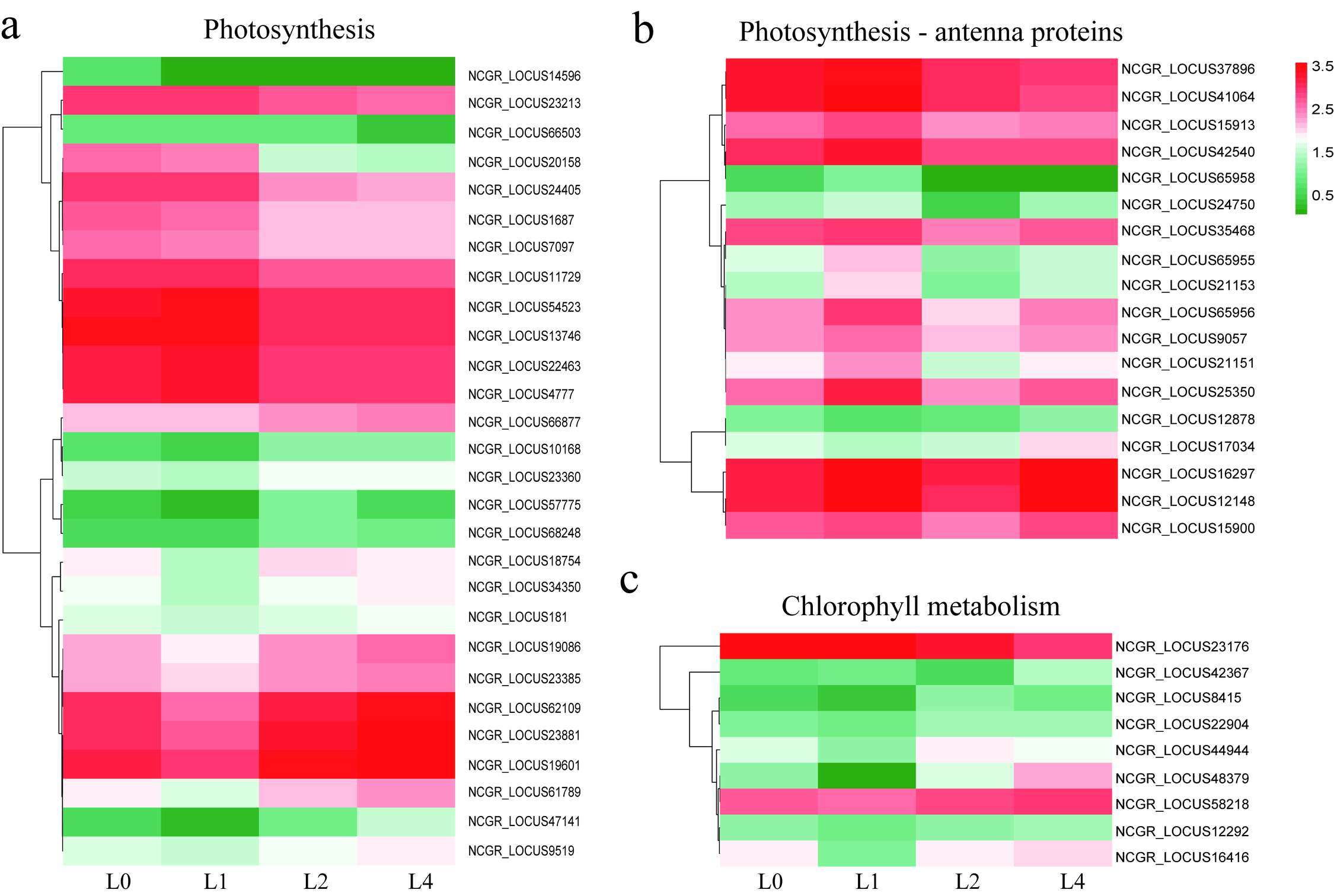

Supplement: S6 Fig — Expression level analysis of photosynthetic system DEGs under the Cd stress, including photosynthesis (a), photosynthesis-antenna proteins (b), chlorophyll metabo lism (c). The red indicates up-regulated expression of DEGs, while green indicates down-regulated expression of DEGs. 0d, 1d, 2d, 4d, days of plants treated with Cd for indicated periods. (TIF) [file pone.0302940.s006.tif]

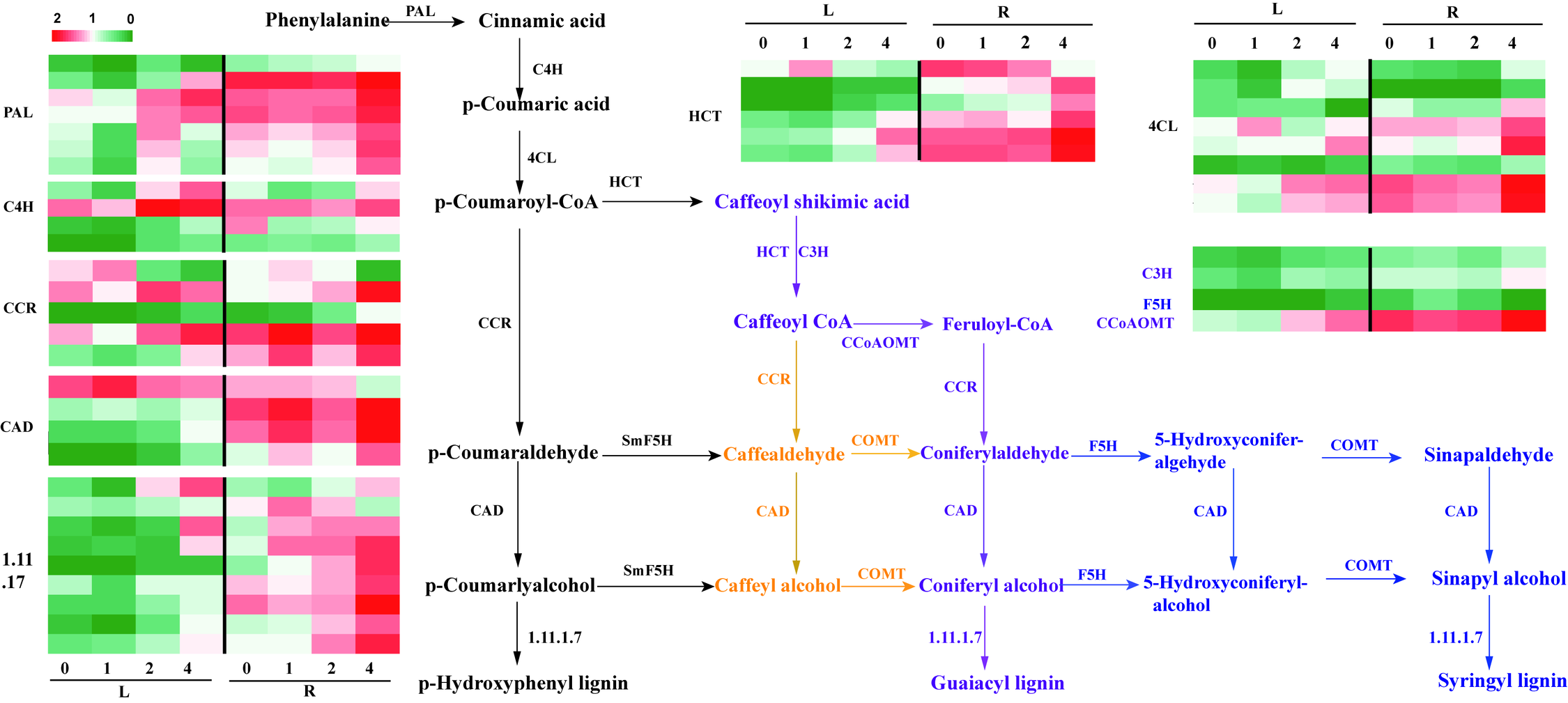

Supplement: S7 Fig — Phenylalanine ammonia-lyase (PAL), cinnamate 4 hydroxylase (C4H), 4-hydroxycinnamoyl-CoA ligase(4CL), cinnamyl alcohol dehydrogenase(CAD), cinnamoyl-CoA reductase(CCR), hydroxycinnamoyltransferase(HCT), Cytochrome P450(C3H), ferulic acid 5-hydroxylase(F5H), caffeoyl-CoA O-methyltransferase (CCoAOMT). The red indicates up-regulated expression of DEGs, while green indicates down-regulated expression of DEGs. 0d, 1d, 2d, 4d, days of plants treated with Cd for indicated periods. (TIF) [file pone.0302940.s007.tif]
