# Supplementary material for: Physiological and transcriptomic analyses reveal the cadmium tolerance mechanism of Miscanthus lutarioriparia
Source: PLoS One. 2024 May 15;19(5):e0302940. doi: 10.1371/journal.pone.0302940 (PMC11095687; doi:10.1371/journal.pone.0302940)
Supplement: Attachment — Submitted filename: Report PONE-D-23-38398.docx [file pone.0302940.s015.docx]

Dear Authors,

I have gone through the manuscript, titled: “Physiological and transcriptomic analyses reveal cadmium tolerance mechanism of *Miscanthus lutarioriparia*”. The work is good, but there are numerous queries throughout the manuscript that should be answered before final decision by the journal Editor.

**Abstract**

The author made a general summary without giving specific details about the results. It's important for them to share the findings using percentages. Also, they should clearly explain the importance of the study in this part. The language of the manuscript is poor and need to be checked by a native speaker before submission of the revised version.

**Line 40-41:** You conclude “These results lay a solid foundation for breeding excellent Cd resistant *M. lutarioriparia* and provide candidate genes for the genetic improvement of other crops.” Even if the crops accumulate more cadmium (Cd) without affecting their yield and other growth aspects; it would still be harmful to humans, isn’t it?

**Line 41:** The word should be “candidate genes” rather “can didate genes”.

The conclusion is not very clear. Please write a clear conclusion.

**Introduction**

In the introduction section, it is important to first state the hypothesis behind the study, outlining the expected outcomes. Following this, the specific objectives of the study should be clearly written.

**Line 70 & 72:** You wrote “*Miscanthus×giganteus*” any specific reason?

Please include more recent references in the list if possible, avoiding those that are over 10 years. old

**M&M**

It's important to know if the soil used in the experiments was sterile.

Where you kept the pots during the experiment?

**Line 108:** “Each treatment was planted with 3 pots, and each pot was planted with 3 plants.” The sentence is so confusing. What do you mean?

**Line 109:** You used the word “natural growth”, what do you mean?

**Line 111:** You checked physiological and transcriptomic effects of *M. lutarioriparia* (Ml004) leaves and roots. Why you excluded stem?

What was the frequency of irrigation?

**Results**

You have mentioned that the cadmium concentration of the soil was increased to 150 mg/kg and the plants ended up with 185.65±15.54 mg/kg (aboveground parts) and 186.80±14.97 mg/kg (belowground parts), explain please.

In hydroponic experiment you have given 18 mg/L of Cd stress, while you ended up 40 mg/kg of Cd in leaves and approximately 100 mg/kg in roots, explain please.

**Line 189:** I reckon it is better to divide the aboveground parts in to leaves and shoot to get a better view of the Cd accumulation.

**Discussion**

Please add those references that are not 10-years old.

**Line 355, 360 & 361:** You wrote “mgl-1, mgkg-1”, it should be written as “mg.l^-1^, mg.kg^-1^”. Check throughout and correct accordingly.

**Overall**

The language of the manuscript is not up to the mark. At many instances the sentences are confusing and should be rewritten. It would be helpful to have a native English speaker or a skilled writer reviews the document before submitting the revised version.
