# Supplementary material for: Physiological and transcriptomic analyses reveal the cadmium tolerance mechanism of Miscanthus lutarioriparia
Source: PLoS One. 2024 May 15;19(5):e0302940. doi: 10.1371/journal.pone.0302940 (PMC11095687; doi:10.1371/journal.pone.0302940)
Supplement: Attachment — Submitted filename: Response to reviewers.docx [file pone.0302940.s016.docx]

1. Please ensure that your manuscript meets PLOS ONE's style requirements, including those for file naming. The PLOS ONE style templates can be found at

https://journals.plos.org/plosone/s/file?id=wjVg/PLOSOne_formatting_sample_main_body.pdf and

<https://journals.plos.org/plosone/s/file?id=ba62/PLOSOne_formatting_sample_title_authors_affiliations.pdf>

**Response:** **We thank the Editor for the professional advices and we revised it in the new version.**

2. Note from Emily Chenette, Editor in Chief of PLOS ONE, and Iain Hrynaszkiewicz, Director of Open Research Solutions at PLOS:

Did you know that depositing data in a repository is associated with up to a 25% citation advantage (https://doi.org/10.1371/journal.pone.0230416)? If you’ve not already done so, consider depositing your raw data in a repository to ensure your work is read, appreciated and cited by the largest possible audience. You’ll also earn an Accessible Data icon on your published paper if you deposit your data in any participating repository (<https://plos.org/open-science/open-data/#accessible-data>).

**Response: We thank the Editor for the professional advices and we revised it in the new version.**

**The database: https://www.ncbi.nlm.nih.gov/bioproject/?term=PRJNA733881**

3. Thank you for stating the following financial disclosure:

"This research was funded by Independent Research fund of Key Laboratory of Industrial Dust Prevention and Control & Occupational Health and Safety (Anhui University of Science and Technology) (NO. EK20202002), the Independent Research fund of Joint National-Local Engineering Research Centre for Safe and Precise Coal Mining (Anhui University of Science and Technology) (NO. EC2021007) and the Postdoctoral Science Foundation of China (2019M662719)." The funders had no role in study design, data collection and analysis, decision to publish, or preparation of the manuscript.

Please state what role the funders took in the study. If the funders had no role, please state: "The funders had no role in study design, data collection and analysis, decision to publish, or preparation of the manuscript." If this statement is not correct you must amend it as needed.

Please include this amended Role of Funder statement in your cover letter; we will change the online submission form on your behalf.

**Response: We thank the Editor for the professional advices and we revised it in the new version.**

**This research was funded by Independent Research fund of Key Laboratory of Industrial Dust Prevention and Control & Occupational Health and Safety (Anhui University of Science and Technology) (NO. EK20202002), the Independent Research fund of Joint National-Local Engineering Research Centre for Safe and Precise Coal Mining (Anhui University of Science and Technology) (NO. EC2021007) and the Postdoctoral Science Foundation of China (2019M662719)." The funders had no role in study design, data collection and analysis, decision to publish, or preparation of the manuscript.**

4. Thank you for stating the following in the Acknowledgments Section of your manuscript:

"We would like to thank the Key Laboratory of Industrial Dust Prevention and Control & Occupational Safety and Health of the Ministry of Education for their experimental platform and technology support. We deeply appreciate the Majorbio I-Sanger Cloud Platform for the help in data analysis. This research was funded by Independent Research fund of Key Laboratory of Industrial Dust Prevention and Control & Occupational Health and Safety (Anhui University of Science and Technology) (NO. EK20202002), the Independent Research fund of Joint National-Local Engineering Research Centre for Safe and Precise Coal Mining (Anhui University of Science and Technology) (NO. EC2021007) and the Postdoctoral Science Foundation of China (2019M662719)."

Please note that funding information should not appear in the Acknowledgments section or other areas of your manuscript. We will only publish funding information present in the Funding Statement section of the online submission form.

Please remove any funding-related text from the manuscript and let us know how you would like to update your Funding Statement. Currently, your Funding Statement reads as follows:

"This research was funded by Independent Research fund of Key Laboratory of Industrial Dust Prevention and Control & Occupational Health and Safety (Anhui University of Science and Technology) (NO. EK20202002), the Independent Research fund of Joint National-Local Engineering Research Centre for Safe and Precise Coal Mining (Anhui University of Science and Technology) (NO. EC2021007) and the Postdoctoral Science Foundation of China (2019M662719)."

Please include your amended statements within your cover letter; we will change the online submission form on your behalf.

**Response: We thank the Editor for the professional advices and we revised it in the new version.**

5. Thank you for stating the following in your Competing Interests section: "NO authors have competing interests"

Please complete your Competing Interests on the online submission form to state any Competing Interests. If you have no competing interests, please state ""The authors have declared that no competing interests exist." ", as detailed online in our guide for authors at http://journals.plos.org/plosone/s/submit-now

This information should be included in your cover letter; we will change the online submission form on your behalf.

**Response: We thank the Editor for the professional advices and we revised it in the new version.**

6. Please note that your Data Availability Statement is currently missing the direct link to access each database. If your manuscript is accepted for publication, you will be asked to provide these details on a very short timeline. We therefore suggest that you provide this information now, though we will not hold up the peer review process if you are unable.

**Response: The direct link of database: https://www.ncbi.nlm.nih.gov/bioproject/?term=PRJNA733881**

**Reviewer #1:**

Dear Authors,

I have gone through the manuscript, titled: “Physiological and transcriptomic analyses reveal cadmium tolerance mechanism of *Miscanthus lutarioriparia*”. The work is good, but there are numerous queries throughout the manuscript that should be answered before final decision by the journal Editor.

**Response:** We are very grateful to the reviewer for the professional advices. The language of this manuscript has been reedited by professional English colleagues. We supplemented related references as support of statement in the introduction and discussion. We also filled some methodological details in this new version. Other questions/concerns that highlighted on the attached document were all revised in the current version.

**Abstract**

**The author made a general summary without giving specific details about the results. It's important for them to share the findings using percentages. Also, they should clearly explain the importance of the study in this part. The language of the manuscript is poor and need to be checked by a native speaker before submission of the revised version.**

**Response:** We are very grateful to the reviewer for the professional advices and we revised it in the new version. We have added some detailed results. The language of this manuscript has been reedited by professional English colleagues.

**Line 40-41: You conclude “These results lay a solid foundation for breeding excellent Cd resistant *M. lutarioriparia* and provide candidate genes for the genetic improvement of other crops.” Even if the crops accumulate more cadmium (Cd) without affecting their yield and other growth aspects; it would still be harmful to humans, isn’t it?**

**Response:** Yes，we strongly agree with your point . But, the crops in the paper are specific plants that can be used for **phytoremediation** (A review on phytoremediation of contaminants in air, water and soil. Journal of hazardous materials, 2021, 403: 123658. ), not food crops that we eat. The aim of the research is to obtain a plant that can be used to repair soil heavy metal pollution.

**Line 41: The word should be “candidate genes” rather “can didate genes”.**

**The conclusion is not very clear. Please write a clear conclusion.**

**Response:** We thank the reviewer for pointing this out and we apologize for mischaracterizing it in our manuscript.

**Introduction**

**In the introduction section, it is important to first state the hypothesis behind the study, outlining the expected outcomes. Following this, the specific objectives of the study should be clearly written.**

**Response:** We are very grateful to the reviewer for the professional advices and we revised it in the new version.

**Line 70 & 72: You wrote “*Miscanthus×giganteus*” any specific reason?**

**Response:** We are very grateful to the reviewer for the professional advices. The Miscanthus×giganteus is a triploid hybrid of a diploid *Miscanthus sinensis* and a tetraploid *Miscanthus sacchariflorus*. So, *Miscanthus×giganteus* is Latin name.

Such as the reference: Evaluation of Miscanthus× Giganteus Tolerance to Trace Element Stress: Field Experiment with Soils Possessing Gradient Cd, Pb, and Zn Concentrations. Plants, 2023,12(7), 1560. Remediation of soils on municipal rendering plant territories using Miscanthus× giganteus. Environmental Science and Pollution Research, 2023, 30(9): 22305-22318.

**Please include more recent references in the list if possible, avoiding those that are over 10 years. old**

**Response:** We are very grateful to the reviewer for the professional advices and we revised it in the new version.

**M&M**

**It's important to know if the soil used in the experiments was sterile.**

**Response:** The soil is not sterile. These soil from the field was air-dried and sieved over a 4 mm mesh before used.

**Where you kept the pots during the experiment?**

**Response:** The pots were kept in glasshouses (day/night temperature 28℃/25℃, light/darkness duration 16 h/8 h, and humidity 60%) during the experiment.

**Line 108: “Each treatment was planted with 3 pots, and each pot was planted with 3 plants.” The sentence is so confusing. What do you mean?**

**Response:** We are very sorry for the confusion caused to you by our writing. This means “all experimental materials are planted in treated pots, and each material is not less than 9 plants”

**Line 109:** **You used the word “natural growth”, what do you mean?**

**Response:** We are very sorry for the confusion caused to you by our writing. This means “Plants grow normally in glasshouses (day/night temperature 28℃/25℃, light/darkness duration 16 h/8 h, and humidity 60%)”

**Line 111: You checked physiological and transcriptomic effects of *M. lutarioriparia* (Ml004) leaves and roots. Why you excluded stem?**

**Response:** We thank the reviewer for the professional advices. Because the root is the first or direct part of the plant to contact Cd, and the leaf is the last part to contact Cd (after the root absorbs it, it is finally transported to the leaf). So, we mainly focused physiological and transcriptional changes in leaf and root tissues.

**What was the frequency of irrigation?**

**Response:** Rinse twice with tap water and three times with sterile water.

**Results**

**You have mentioned that the cadmium concentration of the soil was increased to 150 mg/kg and the plants ended up with 185.65±15.54 mg/kg (aboveground parts) and 186.80±14.97 mg/kg (belowground parts), explain please.**

**Response:** The dry weight of each plant is about 100 g in the experiment, and the total amount of Cd adsorbed is **18.6 mg** based on the concentration of 186 mg /kg. Each pot contained 3kg of soil, the Cd content was **450 mg** based on the concentration of 150 mg /kg. So, the plant adsorb only **4.1%** of the Cd content in the soil.

**In hydroponic experiment you have given 18 mg/L of Cd stress, while you ended up 40 mg/kg of Cd in leaves and approximately 100 mg/kg in roots, explain please.**

**Response:** The dry weight of each plant is about 10 g in the experiment, and the total amount of Cd adsorbed is **0.4-1 mg** based on the concentration of 40-100 mg /kg. The content of Cd in each culture solution (2L) was **36 mg** based on the concentration of 18 mg /L. So, the plant adsorb only **1.1-2.7%** of the Cd content in the culture solution.

**Line 189: I reckon it is better to divide the aboveground parts in to leaves and shoot to get a better view of the Cd accumulation.**

**Response:** We thank the reviewer for the professional advices and we strongly agree with your point of view. But, in this study, we mainly considered to calculate the Cd transfer rate of plants (TF = (Cd concentration in aboveground part)/(Cd concentration in belowground part). So, we mixed leaves and stems as above-ground parts for detection. In the future, we will further analyze the Cd content of the leaves, above-ground stems, underground stems and root tissues of *Miscanthus lutarioriparia* according to the ideas provided from you.

**Discussion**

**Please add those references that are not 10-years old.**

**Response:** We thank the reviewer for the professional advices and we revised it in the new version.

**Line 355, 360 & 361:** **You wrote “mgl-1, mgkg-1”, it should be written as “mg.l^-1^, mg.kg^-1^”. Check throughout and correct accordingly.**

**Response:** We thank the reviewer for pointing this out and we apologize for mischaracterizing it in our manuscript. We reedited the reference in the new version.

**Overall**

**The language of the manuscript is not up to the mark. At many instances the sentences are confusing and should be rewritten. It would be helpful to have a native English speaker or a skilled writer reviews the document before submitting the revised version.**

**Response:** We are very grateful to the reviewer for the professional advices. The language of this manuscript has been reedited by professional English colleagues.

**Reviewer #2: The current study reports on the Physiological and transcriptomic analyses reveal cadmium tolerance mechanism of *Miscanthus lutarioriparia*. Overall, this is a good piece of work with interesting findings. The paper may be accepted for publication after the below minor corrections.**

**Response:** We are very grateful to the reviewer for the professional advices. The language of this manuscript has been reedited by professional English colleagues. We supplemented related references as support of statement in the introduction and discussion. We also filled some methodological details in this new version. Other questions/concerns that highlighted on the attached document were all revised in the current version.

1. The abstract lacks clarity, specific methodological details.

**Response:** We thank the reviewer for the professional advices and we revised it in the new version. We filled some methodological details in this new version.

2. Revise the key words.

**Response:** We thank the reviewer for the professional advices and we revised it in the new version.

3. Abbreviation should be in full form on its first appearance.

**Response:** We thank the reviewer for the professional advices and we revised it in the new version.

4. Line 177-78 to evaluate the adsorption capacity of Cd in soil for *M. lutarioriparia* should be deleted.

**Response:** We thank the reviewer for the professional advices and we revised it in the new version.

5. Line 188 to evaluate the Cd adsorption properties of *M.lutarioriparia*, the content of Cd in treatment group and control group should be deleted.

**Response:** We thank the reviewer for the professional advices and we revised it in the new version.

6. It is better to add some more explanation and references in the discussion section.

**Response:** We are very grateful to the reviewer for the professional advices. We supplemented related references as support of statement in the discussion.

**8.** Some of the references are missing essential details, such as the volume and page numbers for journal articles, publication years etc.

**Response:** We thank the reviewer for pointing this out and we apologize for mischaracterizing it in our manuscript. We reedited the reference in the new version.
